# Recovery after caesarean birth: a qualitative study of women's accounts in Victoria, Australia

**DOI:** 10.1186/1471-2393-10-47

**Published:** 2010-08-18

**Authors:** Michelle A Kealy, Rhonda E Small, Pranee Liamputtong

**Affiliations:** 1 Mother and Child Health Research, La Trobe University, Bundoora, Australia; 2 School of Public Health, La Trobe University, Bundoora, Australia

## Abstract

**Background:**

The caesarean section rate is increasing globally, especially in high income countries. The reasons for this continue to create wide debate. There is good epidemiological evidence on the maternal morbidity associated with caesarean section. Few studies have used women's personal accounts of their experiences of recovery after caesarean. The aim of this paper is to describe women's accounts of recovery after caesarean birth, from shortly after hospital discharge to between five months and seven years after surgery.

**Method:**

Women who had at least one caesarean birth in a tertiary hospital in Victoria, Australia, participated in an interview study. Women were selected to ensure diversity in experiences (type of caesarean, recency), caesarean and vaginal birth, and maternal request caesarean section. Interviews were audiotaped and transcribed verbatim. A theoretical framework was developed (three Zones of clinical practice) and thematic analysis informed the findings.

**Results:**

Thirty-two women were interviewed who between them had 68 births; seven women had experienced both caesarean and vaginal births. Three zones of clinical practice were identified in women's descriptions of the reasons for their first caesareans. Twelve women described how, at the time of their first caesarean section, the operation was performed for potentially life-saving reasons (Central Zone), 11 described situations of clinical uncertainty (Grey Zone), and nine stated they actively sought surgical intervention (Peripheral Zone).

Thirty of the 32 women described difficulties following the postoperative advice they received prior to hospital discharge and their physical recovery after caesarean was hindered by a range of health issues, including pain and reduced mobility, abdominal wound problems, infection, vaginal bleeding and urinary incontinence. These problems were experienced across the three zones of clinical practice, regardless of the reasons women gave for their caesarean.

**Conclusion:**

The women in this study reported a range of unanticipated and unwanted negative physical health outcomes following caesarean birth. This qualitative study adds to the existing epidemiological evidence of significant maternal morbidity after caesarean section and underlines the need for caesarean section to be reserved for circumstances where the benefit is known to outweigh the harms.

## Background

A recent WHO survey of nine Asian countries concluded that to improve maternal and perinatal outcomes, caesarean section should be performed only when medically indicated [1]. A small proportion of caesarean operations are likely to be performed for life-saving or unambiguous reasons [2,3] the indications for which have not changed greatly in the past fifty years [4]. There is, however, an increasing number of caesareans performed either in a 'grey zone' for a range of ambiguous, uncertain reasons [2,5], or for non-medical indications [6,7]. The caesarean section rate in Australia was 21 percent in 1998 and almost 31 percent in 2007 [8]. In England, caesarean section accounted for 24.6% of births in 2008 and 2009 [9]. In stark contrast, Brazil's caesarean section rate varies widely from as high as 70% in the private sector, which provides care to about one quarter of all childbearing women, and 28% in the public sector [10].

Non-clinical factors [11,12] are reported to contribute to the rising caesarean section rate in the developed world and include variation in clinical practice [13], caregiver fear of litigation [14,15], caregiver assessment of risk [16], maternal health insurance and hospital admission status [17-19] and maternal fear of labour and birth [20-22].

Debate about the risks and benefits of elective (planned) caesarean section continue [23-25] in the absence of good quality evidence [26]. Taiwanese women believe giving birth at an auspicious time affords the newborn baby a better life and obstetric providers are prepared to honour women's preference for a caesarean section even though there is an increased risk of an adverse outcome [27]. Planned, pre-labour caesarean section appears to offer more protection to the pelvic floor than an emergency operation in second stage, or an instrumental vaginal birth [28-31]. Scheduling a caesarean in advance affords women and their families, their caregiver and place of birth the convenience of knowing the day and time of birth [32-34]. The rights of women to choose caesarean section over the rights of the fetus have been argued [35], as has the notion that a 'good' mother would choose caesarean if it is portrayed as a safer and more controlled mode of birth for her unborn infant [36].

Caesarean birth is associated with increasing rates of severe maternal morbidity [37], including potentially fatal complications, including sepsis, thromboembolic events, anaesthetic complications [38] and hospital readmission [39]. After adjusting for maternal age, demographic factors and pre-existing medical complications, emergency caesarean section has been shown to quadruple the risk of having a life threatening event; treble the risk of severe haemorrhage, and represents almost twelve times greater risk for severe sepsis [40].

Irrespective of the type of caesarean section, an overall maternal post-operative complication rate of 36% has been reported [41]. Infection is the most common maternal complication after caesarean section and accounts for considerable morbidity and rehospitalisation [42]. There is a three-fold increased risk of puerperal febrile morbidity in women who had a caesarean delivery in labour, compared with women who had a caesarean section without labour [43]. US data showed women who had caesarean birth were more likely to require readmission for uterine infection than women who had assisted or spontaneous vaginal birth [44]. Australian women who had a caesarean birth were more likely to require readmission to hospital for undefined health problems in the first eight weeks after birth, compared to women who had unassisted vaginal births [45]. Physical complications, minor and major after caesarean section, may delay maternal recovery [46].

More than a third of women in Sweden who had a caesarean section reported minor or major problems associated with wound pain four to eight weeks postpartum [47], whilst primiparous women who had a caesarean section were about half as likely as women who had an unassisted delivery to report at seven weeks postpartum that bodily pain had not interfered with their usual activities in the previous four weeks [48].

In an international trial of mode of delivery for term breech, women randomised to planned caesarean section were 25% more likely to suffer serious morbidity using a composite measure during the first six weeks postpartum than women assigned to planned vaginal delivery [49]. In Australia, 63% of women who had an emergency caesarean section and 59% of women who had an elective caesarean section reported wound pain at six to seven months postpartum [30]. Six years after only ever having caesarean section, 14% of women still reported persistent urinary incontinence, despite surgical birth [50].

The health of mothers after caesarean section has been described as a particular area of neglect [51]. Minor health problems after childbirth are frequently under-reported to health professionals [52] and sometimes primary care providers do not discuss common postnatal problems with women [53]. Prolonged maternal recovery, restricted mobility and other minor health problems after caesarean section have also been reported [54]. Anecdotally, routine post-operative advice given to women after caesarean section prior to discharge from hospital includes the avoidance of heavy lifting and driving a vehicle and abstinence from sexual intercourse for approximately four to six weeks. However, this advice, while it may be well-meaning and supportive of new mothers in need of rest and recuperation, appears to be without a solid evidence base.

To date the majority of studies examining maternal morbidity following caesarean birth have used linked birth certificate or other population-based data or medical record review [38,43,55]. The aim of this paper is to describe women's accounts of recovery after caesarean birth, from shortly after hospital discharge to between five months and seven years after surgery.

## Method

### Participants

Interviews were conducted from December 2003 to December 2005 with women who had previously enrolled in a randomised trial (RCT) of midwife-led debriefing after operative birth at a major tertiary hospital in Victoria, Australia [56] and had completed a four to six year follow-up postal survey [57] in which they indicated they were happy to participate in future research. Women who had births prior or subsequent to their operative birth may have done so at a hospital other than the tertiary hospital in which they were recruited for the trial.

Thirty-four women, all of whom had at least one caesarean, were telephoned using a contact protocol, and 32 agreed to take part (one woman was due to give birth for the fourth time; one woman declined participation). All women resided in metropolitan, regional or rural Victoria.

### Design and procedures

The personal interview, one of the most common approaches to qualitative research in the public health literature, offers an opportunity to interact with study participants during data collection [58]. This study was designed to better understand women's experiences of caesarean section including their immediate and longer term recovery. Background information to the study and consent forms were posted to women in advance of the interview. MK conducted one in-depth face-to-face interview with each woman at a time and place of her choice. Participation in the study was entirely voluntary and each participant understood they were free to withdraw at any time. All interviews were audio-taped and transcribed verbatim by MK.

An interview topic guide [59] acted as a prompt to ensure important milestones in women's pregnancy and birth experiences were explored, however, women were encouraged to lead the conversation and share what was important to them. The focus of the interviews was to sensitively glean insight into women's views of childbirth, the role of women and caregivers in decision-making regarding mode of birth, and women's experiences of recovery after becoming a mother. Each of the interviews followed a similar direction irrespective of women's mode of birth Field notes were recorded after interviews to capture additional conversation or events that were not collected on audio-tape. Interviews averaged approximately 80 minutes.

Sampling for interviews began pragmatically. Women who had a recent caesarean birth (within the previous 12 months) were interviewed first to capture their views and experiences of their recovery in the shorter term (n = 7). Interviews were then undertaken with women who had only ever had one birth and by caesarean (n = 6) to learn about women's longer-term recovery. The sample was then further diversified to interview women who had a vaginal birth before or after a caesarean (n = 8), or more than one caesarean and had never had a vaginal birth (n = 5). The next phase of sampling focused on recruiting women who had actively sought a caesarean (n = 6), as only two women interviewed to that point were in this category. This approach ensured a diverse range of women's experiences of caesarean birth were included in the study [60].

The study was approved by the Human Ethics Committees of the tertiary hospital where women were recruited for the original study, and La Trobe University, Bundoora, Australia.

### Analysis

Transcription and preliminary data analysis were undertaken contemporaneously to check for emerging and recurring concepts. MK and RS read and discussed the transcripts and highlighted potential key themes to be explored in future interviews. Thematic analysis continued up to the completion of all interviews and new ideas were explored and checked against the data as themes emerged from women's diverse accounts [61].

The reasons and the decision-making process which women described for their first caesarean section were grouped into three categories (called three Zones of Clinical Practice), in order to understand whether the reasons for the caesarean impacted on women's experiences of recovery. This became the theoretical framework for the study. Central zone caesarean sections are undertaken for unambiguous life-saving reasons. The benefits of the surgery are known to outweigh the potential for any harm; the surgery is essential. Grey zone caesarean sections are undertaken in the presence of clinical uncertainty about the potential for benefit compared with the potential for harm. Peripheral zone caesarean sections are undertaken for non-clinical reasons (maternal request) and the potential for harm is thus likely to outweigh the potential for benefit.

## Results

### Socio-demographic characteristics

Socio-demographic details of study participants according to the three zones of clinical practice are summarised in Table 1. Twelve women described how their first caesarean was undertaken for potentially life-saving reasons (Central zone). Eleven women described clinical uncertainty (Grey zone), and nine women stated they actively sought surgical intervention (maternal request) in the absence of clinical indications (Peripheral zone) at the time of their first caesarean section (Table 2). There were a total of 67 confinements (68 births) with 50 caesarean births and 17 vaginal births in the sample. There was a mix of planned and unplanned surgical procedures and instrumental and unassisted vaginal births.

**Table 1:** Interviewee characteristics at time of first caesarean birth

	**Zone of clinical practice (1**^**st **^**caesarean)**		
	Central zone n = 12	Grey zone n = 11	Peripheral zone n = 9
**Age**			
20-24yrs	1	0	0
25-34	8	8	6
35-39	1	3	2
40-42	2	0	0
**Admission status**			
Public	11	7	6
Private	2	4	2
**Parity at 1**^**st **^**caesarean**			
Primiparous	11	9	3
Multiparous	1	2	9
**Educational attainment**			
Secondary	5	5	4
Further education, incl. tertiary studies	7	6	5
**Employment status**			
Student	1	0	1
Full time employment	12	11	7
Part-time employment	0	0	2
**Marital status**			
Married	8	11	7
De Facto	3	2	1
**Place of residence**			
Metropolitan	10	11	7
Regional/Rural	1	2	1
**Country of Birth/1**^**st **^**language other than English spoken**			
Australia	8	11	3
South East Asia	1	0	1
UK/NZ	3	0	4
Other European	0	0	1
1^st ^language other than English	1	0	2

**Table 2:** Summary of three Zones of Clinical Practice

Zone	Explanation	Examples from women's accounts
Central zone	Caesarean section performed for unambiguous clinical reasons* (life-saving)	Footling breech, hand presentation, severe oligohydramnios at term, not in labour; severe pre-eclampsia; antepartum haemorrhage in labour, prior caesarean; severe unstable asthma.
Grey zone	Caesarean section performed for ambiguous clinical reasons*	Maternal 'exhaustion'; 'slow' progress in labour; mild gestational diabetes; suspicion of 'big' baby; mild hypertension.
Peripheral zone	Caesarean section performed in the absence of clinical reasons	Maternal request (past history of negative birth experience, fear for baby's safety, fear of vaginal birth).

*Adapted from: *A guide to effective care in pregnancy and childbirth *ed. Enkin M, Keirse M, *et. al*. (2000); Oxford University Press, Oxford [2].

The physical health issues that women experienced after caesarean birth were diverse. Almost all women (30/32), irrespective of the reason for their caesarean, described at least one complication or health problem related to surgical childbirth.

### Being a 'good mother': the difficulties of following postoperative advice after caesarean birth

Women described how prior to discharge from hospital they were given routine post-operative advice to follow for at least four to six weeks after their caesarean. This advice included avoiding any behaviour which might cause abdominal muscle strain, additional pain or wound breakdown. Heavy lifting (such as a full laundry basket), stretching arms high above one's head (necessary to peg clothes on an outside clothes line), driving the car and engaging in sexual intercourse were some of the activities women reported they were advised to refrain from in the short term. Women generally felt frustrated by the physical restriction that caesarean birth imposed. It was an unanticipated negative consequence of surgical childbirth, even if they felt well prepared for the post-operative period. Women were also inclined to feel guilty when they acted against advice in order to accomplish routine infant care and domestic tasks.

Zone of clinical practice for first caesarean is shown after each quote. Pseudonyms are used to protect study participants' identity.

Carol and her husband lived on a farm approximately 400 km from the tertiary hospital where she had an emergency caesarean at 34 weeks gestation. She described how she and her husband started the long drive home after being discharged from hospital, two weeks after her first baby was born:

I drove out of [the city] 'cos my husband doesn't drive in [the city]! He started off driving and I couldn't tell you where we ended up. We ended up somewhere where we weren't supposed to be! And I'm just saying, "Just pull over, just pull over." 'Cos he's getting really frustrated... 'Cos he's used to being in the country I suppose. So of course we missed whatever turn offs and so I ended up changing and I drove. And I said, "Well there went that rule." [Central zone]

Once Carol was at home she had no choice but to drive, even though she was doing it against advice because she was responsible for providing for her family:

It's hard, because they say, don't they, not to drive...for whatever length of time... I live 20 minutes from town, so if I don't drive then we're not going to have milk and bread on the table. [Central zone]

Being unable to obey the rules, as described by Carol in her first quote, caused women concern. Kara said:

You get that advice and if you follow that advice that's good. ... I did have to drive and do extra things. ... I was worried, but there wasn't much I could do, I had to do it. There was no one else. [Grey zone × 2]

Like most women in the study, Leonie had little support at home:

The health nurse that comes and visits...said, "Now, you're not driving?" ... "And you're not hanging the washing on the line?" ... I just said to her, "No, no, no, I'm not doing any of those things." But how else was it going to get out there? Because, [my partner] was working, there's only so much he can do. I'm not going to wait until he gets home to hang the washing on the line. [Grey zone × 3]

Rose had little assistance when her first baby was born. Her husband did not provide her with emotional or physical support and her family and friends lived too far away to offer any practical help. Rose remarked:

The first time I came home and, my husband at that time had been sick that week. And the house was an absolute shambles and hadn't been vacuumed, and that. And the first thing I did when I walked in was vacuum the house! [Peripheral zone]

Rose's personal circumstances offered little choice in terms of tasks such as vacuuming, even though she knew it was contrary to the advice she had received.

In contrast to women who felt they had no choice but to get on with the domestic chores, Bridget left management of her home and care of the baby in the hands of her partner. Bridget contrasts her recovery experiences; the first time living in the city and the second in a country town. She said:

They told me that I wasn't allowed to do anything for six weeks; like no heavy lifting or that sort of thing for six weeks. So [my partner] was basically mum to [our son] for the first six weeks. He wasn't employed at the time. ...He did everything for me, he went and he'd done the housework, done all the washing, set the baby's room up; he did absolutely everything for me. But it was a strange experience like 'cos we did it all on our own. Since having [our second] we've moved down here and a lot of the time I've had help from mum and other friends and family and stuff and it's a big difference from what it was like in [the city] 'cos we did everything ourselves. We knew two people and that was it. And yeah, so we didn't have any help from anywhere. [Central zone + VB × 2]

Women were critical of the advice they received prior to discharge from hospital. Almost always they were the main caregiver to their newborn. They typically had other children to look after and sometimes relatives required their assistance. On the whole, women continued to provide meals, chauffeur older children to school, medical appointments and child care; did the shopping and attended to the bulk of the domestic responsibilities. While they recognised that their behaviour was contrary to the advice they received, women believed they had little or no choice. Their primary role and responsibility was to be a good mother to their newborn and other children and to be a good partner, daughter or sister, depending on individual circumstances. Rarely were women the central recipients of care (particularly beyond the first few days at home) from family members or others in the first six post-operative weeks.

### Unexpected pain and reduced mobility

Women described short and longer-term pain after caesarean section. Performing normal activities of daily life required considerable effort and time. Women described receiving conflicting advice from various health professionals about safe medications when breastfeeding, and so, to avoid harm, opted instead to go without or to take sub-therapeutic doses. Some women could compare their recovery after both a caesarean and a vaginal birth.

Gretel's first baby was born by caesarean and her second was a vaginal birth. She compared her experiences of recovery after childbirth:

It wasn't a particularly wonderful time, you know, looking back on it. I think, you know, I mean, you survive...it took me 13 weeks to recover...I felt completely exhausted most of the time and in a lot of pain. ... [I] sat in front of the fire with a hot water bottle to my cut because I was in pain. You know, not feeling particularly good about anything really...I felt pretty miserable. Whereas with [my second, a vaginal birth] I just felt completely normal. [Central zone + VB]

Prue was disappointed in her reduced capacity to care for her newborn after a caesarean, compared to an earlier vaginal birth:

The physical recovery was so much longer and I don't think you're prepared for that. You're not told that it [a caesarean] is more painful, and that you are in more pain, and its restrictive pain. Like there's things you just can't do. Well, I couldn't do. The bending, the picking up, and things that really are so significant when you have a baby. [VB + Grey zone]

Sarah's second baby was five months old but she still did not feel recovered enough to do everyday chores:

I felt a lot of pressure because I had my older daughter starting school. So I was thinking I need to be able to drive and get around and be more active. And I think this time, after the second, I tried to do too much too soon, to my own detriment really. [Grey zone + Central zone]

Lee Lin suffered different aches and pains after her first caesarean:

With Kylie I remember after birth I had backache, I had it for quite a while. And I have to, when I breastfeed, I have to put a lot of pillows? at my back for comfort. [Central zone ]

After her second caesarean, Lee Lin checked with her general practitioner at six weeks postpartum about a different pain:

I had a pain from my ribs touching my back occasionally. ... I think that went for at least five to six months. ...I told her [GP] about the pain on my rib...she said it's okay. So I didn't worry about it. [Central zone + Peripheral zone]

Lee Lin felt troubled by the pain for a considerably longer time than would be expected to be normal after a caesarean.

Not all women talked about experiencing ongoing pain or reduced mobility for an extended period after caesarean birth.

Amanda had a vaginal birth and seven years later a maternal request caesarean. Her second child was seven years old at the time she was interviewed. She recalled:

I was fully recovered, completely, for both of them, by the six week check. I was cleaning the house the day after I came from hospital with no, you know, discomfort. A little bit achy maybe by the end of the day but, I mean, I didn't overdo it either. I wasn't some stupid woman climbing up ladders or anything. I just pottered around and did my thing. [My partner] did the picking up for school so I didn't have to do all that... I didn't do anything stupid, you've got stiches, you don't want to be picking up anything really heavy, but nobody told me not to. I think its common sense not to. [VB + Peripheral zone]

Amanda described an uncomplicated recovery after her only caesarean. Nevertheless, she behaved conservatively during the postoperative period; she restricted her daily activities and allocated tasks such as driving to her partner.

### Abdominal wound complications

Women's accounts of problems with their abdominal wound were diverse, irrespective of the reason for their caesarean, and whether it was a planned or unplanned procedure. Issues ranged from slight inflammation that did not require any extra care, through to wound breakdown and readmission to hospital. Scarring after wound infection was unsightly and disfiguring. Women rarely understood why there was altered sensation (numbness, itchiness, heightened sensitivity) of the skin near their abdominal scar. When medical review was sought, the explanation women received was usually unsatisfactory and did not allay their concerns. These issues sometimes remained unresolved for several years. For example, Carol said:

It [the scar] used to be tender for quite a while, quite a long time it was quite tender. So I couldn't stand anyone touching me, even the kids. They don't mean to. They might just put their knee into you or something and particularly if it was just on that side. But that's all settled down now. Oh well, [my third] is coming up two years in July so it would want to be settled down or you'd be going back to say there's something really not right. [Central zone + Grey zone]2].

Stacey was reminded of the slight wound infection she had experienced at the time of her first caesarean when she gave birth to her second baby. She did not realise how serious the consequences of a wound infection could be for a new mother. Stacey recounted the story:

The first one [CS] it seemed to be a little bit sore and she [the midwife] said it was a bit red. It didn't get severely infected or anything like that. When I was in hospital with [my second] a girl came in who they put into hospital because her wound was infected, but it wasn't anything bad like that. [Central zone + Grey zone]

Two women feared for their lives when problems arose with their wounds. Sarah described what happened to her on the day she left hospital after her second caesarean. She was home alone with her newborn and four year old daughter as her husband had been unable to take time off work:

I hung out about three loads [of laundry] and that night I could hardly move. And the next day I had a lump come up on the suture line and that started bleeding. So I rang the doctor 'cos I thought I was going to die! And he said, "Look, it should be okay. I'll see you tomorrow though, to check it out." [Grey zone + Central zone]

Gianna's account was even more dramatic:

I came home on the Thursday; on the Friday I started to get really sick and on the Saturday I was almost dead. ... I didn't know that it'd split 'cos it wasn't like a raw split...but it was just pouring with fluid. And we went straight to the hospital, to the emergency, and I was put on a drip straight away...I was in hospital another week. [AVD + Peripheral zone)

Another unexpected negative consequence of an infection post-caesarean was a disfiguring scar. Corrective surgery was one solution offered to women. Prue said:

After both caesars I got infections...I had quite a crooked scar because of the infection I got. ...He did give me the option to come back and have it fixed... Having had the infections after and the healing process of a scar like that; I wouldn't want to go through it again. [SVD + Grey zone × 2]

Surgical revision of her scarring was not a solution Prue was prepared to consider.

Denise's account exemplifies women's lack of information about some of the unanticipated consequences of caesarean. She said, five years after her second caesarean:

Even now, my stomach, once you've been cut, you can't feel anything for a few years at least...the feeling is just not quite there. ...It's always itchy...but you can't satisfy the scratch, 'cos it's almost on the inside of your tummy. And even now I still experience that. [Grey zone × 2]

Leonie's third caesarean was ten months previous and she described still feeling anxious during sexual intercourse:

I do have to say to him [partner] occasionally don't do that, that hurts. And it's not so much, it's not like sharp pain or anything like that, but uncomfortable and it is definitely the scar area.... But that's even now that it's totally healed, that could happen today. [Grey zone × 3]

Anya had an emergency laparotomy six weeks after her only caesarean seven years earlier. She said:

I had quite a lot of skin numbness which I did see someone about quite a long time after the second operation, because I had no sensation on the skin and I thought, gee, this is very peculiar. And I was told, "Well, basically there isn't too much to do about it. And don't worry about it". [Peripheral zone]

In contrast, some women did not experience any wound complications. Wendy recalled her only birth, by caesarean seven years earlier:

[I] had no complications with the surgery, no complications with the sutures, the wound or anything like that. It actually healed up very, very quickly with no problems at all. In fact, the midwife, I remember her saying, "Oh, who did this? It's a really nice job." [Grey zone]

### Vaginal bleeding

The women in this study commonly thought that vaginal bleeding after caesarean section settled quickly and was less compared with vaginal birth. However, for some women ongoing vaginal bleeding had devastating consequences. Medical or surgical intervention was sometimes necessary to treat the cause of bleeding or to manage unpleasant symptoms.

Anya's account was the most dramatic of all the women interviewed who described problematic vaginal bleeding after caesarean section. She recalled what happened to her after she visited her GP and was advised she would need to be readmitted to hospital:

My bleeding didn't stop. So that was about six weeks later; I needed to go back in. I had a D & C [sic] at that stage, which went wrong. It was botched, and I had a punctured uterus and punctured bowel, which needed abdominal surgery to repair. ...So then I was in for I think abut a fortnight after that... I had a belly full of wound. [Peripheral zone]

In contrast to Anya's terrifying surgical experience (a rare outcome), all Therese needed was reassurance over the telephone from her doctor:

I kept bleeding for over six weeks after the caesar... I've rung [my doctor] and said I was still bleeding. ...I had to wear a pad. ...The doctor said sometimes you can have an infection and that keeps the bleeding going. [AVD + Peripheral zone]

Unlike Therese, Mandy needed medical intervention to manage her bleeding. She described how this impacted negatively on her wellbeing:

I had a large bleed afterwards, when I got home. I had to go on antibiotics 'cos they thought maybe I had an infection, maybe a tiny bit of placenta had been left behind. And that sort of didn't help at home, made you sort of feel a bit sick. [2 × Grey zone]

Loress also suffered from the unexpected consequences of ongoing vaginal bleeding after her third caesarean. She describes receiving two kinds of therapy to stop the bleeding and restore her health:

I went to the hospital and I told them I'm still bleeding and they did an internal. And I had to have an internal ultrasound... And I had to go on...oestrogen I think it is...and I had no iron, at all, my iron was depleted completely. [Grey zone × 3]

### Urinary incontinence

Eleven women in this study mentioned having some urinary incontinence either during pregnancy or after childbirth (or both), and four of these women had only ever given birth by caesarean section. The following accounts are from the women who had only ever had caesarean births, either prior to the onset of labour or in the first stage of labour.

Leonie had three caesarean births; the first in early labour and the next two caesareans were planned procedures prior to the onset of labour:

They say you can get incontinent when you get older. So I'm a bit conscious of that, thinking I don't want that to happen to me. I don't know if it was really with the others, but during my third pregnancy, I did have some problems where I do a little bit of wee in my pants and things like that. If I laughed too loudly suddenly, or moved suddenly... [Grey zone × 3]

Denise said urinary incontinence began after her second baby was born. Her first labour did not progress and her second baby was a scheduled operation:

Every now and then, I do notice it where...I might have a dribble for example, and think, oh gosh, I need to work on these muscles a little bit more to get that contraction, or holding on feeling, but, um, not that much. ...I was getting a bit concerned there, thinking, oh, no, don't tell me I'm going to have this problem now. [Grey zone × 2]

Mandy also said her problems began after her second baby. Her first baby was born after failure to progress in the first stage of labour and her second was a planned caesarean:

I was coughing so badly that I did have some slight leakage happening - for the first time ever - and just fractionally, a couple of times. And, oh, what's wrong, I'm not meant to be having this, not that I've been doing any exercises though, not that I thought I needed to, but I didn't have any problems after the first, it was just now with this terrible cough. [Grey zone × 2]

## Discussion

This paper offers insights from women themselves on recovery after caesarean section. Strengths of the study include the diversity of the sample, with women who had more than one type of birth, and women who had a recent experience of a caesarean and were still recovering from the operation. In addition, women's accounts provide rich and diverse descriptions of their thoughts and experiences. When examined within a theoretical framework - in this study, a typology of decision making - and the existing literature, the findings can make a useful contribution towards informing clinical practice [62]. The study is limited to women who spoke English, women were interviewed on one occasion only and for some women, this was a number of years after giving birth. However, women's recall of their childbirth experience has been reported to be accurate for many years after the event [63].

Thirty of the 32 women spoke at length about the physical difficulties they encountered after caesarean birth, irrespective of the reason for the surgery. At the time of their first caesarean, women were largely unaware of the range of medical and surgical complications that can be associated with major abdominal surgery. Maternal recovery was complicated by pain and reduced mobility, abdominal wound issues, vaginal bleeding and urinary incontinence.

Prior to discharge from hospital women who have a caesarean section are given routine post-operative advice about the importance of resting and making time to recover. This is the same advice given to patients after any major abdominal surgery. Women are advised to avoid any activity, such as rigorous exercise and heavy lifting which might strain their stitches and abdominal muscles until the healing process is complete. This advice was almost always ignored by the women in this study, who, once at home, had little choice but to assume the majority of caring work for the family [64]. This was particularly true for those women who had older children at home. Women understood they should avoid lifting heavy loads of laundry, refrain from lifting the toddler and desist from driving. They also knew that in order to care for the baby's and family's needs they must undertake these 'prohibited' tasks. The advice women receive prior to discharge from hospital after caesarean section needs to be revised to consider the context of women's and their families' lived reality.

On the whole, women knew that the post-operative advice they received conflicted with fulfilling their important role as new mothers. When questioned by health professionals, women felt compelled to deny the extent of their physical activity for fear of censure. If postoperative complications developed, new mothers felt guilty for requiring additional medical attention, and for feeling unable to take complete care of their newborn. Mothering has been described as a private affair until something goes wrong and then mothers are subjected to public scrutiny and criticism [65].

Women in this study described their struggles to balance finding opportunities to rest to avoid pain, exhaustion and wound breakdown, while still providing the majority of infant and child care and home maintenance in the weeks and sometimes months after having a caesarean section. It has been argued that the 'good' mother is a social construct that runs counter to the lived reality of most mothers [66]. Women would have liked to comply with the advice they received from their health professionals because 'good' mothers follow professional advice so as to not jeopardise their recovery [67]. However, the reality was that women could not care for and nurture their own baby, or attend to domestic responsibilities if they followed the postoperative advice they had been given. As the women in this study said, if they could follow the rules or advice given, it would be good, but they did not feel they realistically could.

Few women were prepared for the wide range of physical consequences of birth by caesarean, and were both surprised and frustrated when pain hampered their day to day activities beyond four to six weeks. These findings add to the existing Australian and international population-based evidence of longer maternal recovery time after caesarean [30,46,48].

Women in this study reported a range of wound complications which occurred in the days and weeks after caesarean which were unwanted, unexpected and sometimes debilitating. The extent of the problem varied from minor to major complications, with two women requiring readmission to hospital. The rate of wound problems after caesarean section was recently reported to be almost 14% in the United Kingdom (the incidence ranged from almost three to almost 18%), with 84% of problems detected after discharge from hospital and almost nine percent surgical site infections [68].

Vaginal bleeding after caesarean section is most likely caused by endometritis, an inflammation of the uterine lining. It is up to ten times more common after caesarean section and may occur within 48 hours of birth or up to six weeks postpartum [69]. Major puerperal infection is one of the main reasons for maternal readmission and is associated with shorter lengths of stay after caesarean [70]. A recent Cochrane review compared the use of prophylactic antibiotics with no prophylaxis and found antibiotics to be effective in reducing the incidence of wound infection and endometritis. While the review showed evidence of benefit for women, the potential for adverse effects for mother and baby remain uncertain [71].

A few women who had only ever had a caesarean birth, either before the onset of labour or in the first stage of labour, mentioned experiencing urinary incontinence. This might be seen as a surprising finding. One of the beliefs about planned caesarean section is that it offers protection to the pelvic floor [72]. However a recent study compared women who had caesarean and vaginal births and found 35% of women had urinary stress incontinence after caesarean section, which though lower than the vaginal birth group (54%) was still a significant proportion [73].

## Conclusion

From the personal accounts of women in this study, and the now abundant evidence relating to significant maternal morbidity after caesarean section, the postoperative advice given to women prior to discharge from hospital after caesarean section needs to be reconsidered. This study contributes to the gap in our knowledge about the lived reality for mothers after caesarean section. Women's reports of their physical recovery after caesarean section highlight significant and from women's perspectives, largely unexpected morbidity extending weeks and months after birth. These difficulties experienced by women may seem less critical when the caesarean surgery is life-saving. However, these maternal health and recovery issues need to be taken more seriously in the context of increasing numbers of women having caesarean sections in the presence of uncertain, or no clinical indications, and underlines the importance of efforts to reduce non-medically indicated caesarean section. When caesarean section is performed where the evidence of benefit is not clear-cut, or when no clinical indication exists, then it is paramount that women and their caregivers are clearly informed about, and have a good understanding of all the potential sequelae.

## Abbreviations

MK: Michelle Kealy; RS: Rhonda Small; VB: vaginal birth; AVD: assisted vaginal delivery

## Competing interests

The authors declare that they have no competing interests.

## Authors' contributions

RES conceived the study, MAK and RES designed the study, MAK collected and transcribed the data; MAK with assistance from RES & PL analysed and interpreted the data; MAK drafted the manuscript; MK, RS & PL revised the manuscript; all authors gave final approval.

## Pre-publication history

The pre-publication history for this paper can be accessed here:

http://www.biomedcentral.com/1471-2393/10/47/prepub
